# Impact of Bulk ZnO, ZnO Nanoparticles and Dissolved Zn on Early Growth Stages of Barley—A Pot Experiment

**DOI:** 10.3390/plants9101365

**Published:** 2020-10-15

**Authors:** Lucia Nemček, Martin Šebesta, Martin Urík, Marek Bujdoš, Edmund Dobročka, Ivo Vávra

**Affiliations:** 1 Institute of Laboratory Research on Geomaterials, Faculty of Natural Sciences, Comenius University in Bratislava, Mlynská dolina, 842 15 Bratislava, Slovakia; martin.sebesta@uniba.sk (M.Š.); martin.urik@uniba.sk (M.U.); marek.bujdos@uniba.sk (M.B.); 2 Institute of Electrical Engineering, Slovak Academy of Sciences, 841 04 Bratislava, Slovakia; elekdobr@savba.sk (E.D.); ivo.vavra@savba.sk (I.V.)

**Keywords:** agricultural soil, bioaccumulation, chernozem, nanoparticles, toxicity, zinc oxide

## Abstract

Zinc is among the most in-demand metals in the world which also means that a considerable amount of this element is released to the environment each year as a result of human activities. A pot experiment was conducted to study the impact of low- and high-dose zinc amendments on plant growth and biomass yield, with Calcic Chernozem as a growing medium and barley (*Hordeum vulgare* L.) as a model plant. The distribution of zinc in various plant parts was also investigated. Zn (II) was added in powder as bulk ZnO and in solution as ZnO nanoparticles and ZnSO_4_ in two dosages (3 and 30 mmol kg^−1^ soil) prior to planting. The plants were harvested after 10 days of growth. The three sets of data were taken under identical experimental conditions. The application of zinc in aqueous solution and in particulate form (having particle sizes in the range of <100 nm to >500 nm) at concentration of 3 and 30 mmol Zn kg^−1^ to the soil resulted in decreased growth (root length, shoot length) and biomass yield; the only exception was the addition of 30 mmol Zn kg^−1^ in the form of bulk ZnO, which had a positive effect on the root growth. The dry weight reduction (sprout biomass) was lowest in plants grown in soil treated with dissolved zinc. There were no statistically significant changes in the content of chlorophyll a, chlorophyll b*,* and total chlorophyll, although flame atomic absorption spectrometry (FAAS) analysis indicated that plants bioaccumulated the zinc applied. This implies that the transport of zinc into the above-ground plant parts is controlled by the presence of effective mechanical and physiological barriers in roots. Crop performance under zinc stress in relation to biomass production and the growth of roots and shoots is also partly a reflection of the effects of soil properties. Our findings emphasize the importance of considering plant-soil interactions in research of potential toxicity and bioavailability of zinc in the environment.

## 1. Introduction

Small size and, consequently, the high surface-to-volume ratio of engineered nanoparticles (NPs) often offer advanced or novel properties compared to their bulk counterparts. Engineered NPs are also of a more regular shape, size, and homogeneous composition than those from natural sources [1,2] which makes them well suited for use in a variety of industrial and commercial applications. Zinc oxide (ZnO) material has been used in industry for decades, experiencing a scientific and technical renascence in the last years [3]. The global ZnO market size is estimated to reach USD 5.7 billion by 2024. Besides rubber, which was the largest application of the ZnO market in 2018, this white inorganic compound has also been extensively used in ceramics, cosmetics, pharmaceutical, chemical, and glass industries [4]. It is being applied as a catalyst or sorbent in desulfurization processes, in drilling fluids, varistors, solar cells, textiles, and it has even found its place in agriculture and food production. ZnO nanosized counterparts appear to be a better alternative in many commercial processes where a combination of small size and high surface area presents an advantage. Using nanosized ZnO reduces the total amount of applied ZnO, offers an opportunity to engineer materials with enhanced properties and enables development of innovative applications in all fields of expertise [5,6].

Considering high production and estimates of numbers of engineered particles released into the environment either intentionally or unintentionally, arable land is deemed to be at a high risk of exposure to nanomaterials. A large portion of fabricated NPs get into soil as a result of the use of biosolids which are delivered from sewage treatment plants and animal husbandry facilities and are widely accepted as fertilizers. For instance, ZnO is commonly added to swine diets to prevent post-weaning diarrhoea and bowel oedema in piglets. Literary sources usually report doses within 2000–4000 mg kg^−1^ range; in most European Union countries, the maximum recommended pharmacological concentration of elemental zinc in final medicated feed mixture is 2500 mg kg^−1^. Such supplements may pose a risk to the environment, because dietary ZnO is eventually excreted in feces due to its low absorption efficiency [7]. When rapidly decomposable organic matter, such as manure consisting of animal feces and urine, is added to soil, zinc may become more available due to the formation of soluble organic zinc complexes which are mobile, easily transported and available for absorption by plant roots [8]. On the other hand, ZnO solubility in soils decreases with increasing soil pH making ZnO nano- and microparticles less accessible to plants. Furthermore, the transport of these particles into above-ground plant parts may be limited due to the presence of effective mechanical or physiological barriers in roots [9].

Owing to the physical and chemical properties of zinc (it is not volatile unless heated to very high temperatures and does not degrade), continuous application of manure from medically-treated animals to cropland will result in a gradual increase in zinc concentration in the upper soil layer followed by an increase in zinc content in other significant components of the environment. Thus, it is only a matter of time before the limit values in any of these components are exceeded. For this reason, in 2017, the European Commission voted in favour of a ban on the veterinary use of ZnO. It has been concluded that the benefits of using ZnO do not outweigh the disadvantages. Veterinary medicines containing this compound are planned to be phased out at European Union level by 2022. However, since it is still being viewed as a potential micronutrient input, the future is not likely to bring a decrease in a volume of both direct and in-direct applications of nano ZnO. Therefore, identifying pathways and rates in which the elements in biosolids from industrial, agricultural and urban sewage re-enter the food chain is of utmost importance.

The focus of this paper is on the impact of different forms of ZnO (dissolved, nanosized and bulk) on plants serving as initial energy sources for primary consumers at the bottom of a food chain, using barley as a model plant. Barley is a versatile crop, used both for human consumption and animal feed, and has traditionally been considered an excellent experimental model organism allowing advances in plant genetics, plant physiology, plant pathology, plant biochemistry and more recently in plant biotechnology [10]. The goals of this study were three-fold: (i) evaluate changes in root and shoot length and weight of barley exposed to bulk ZnO, ZnO-NPs and dissolved zinc after the first ten days of growth in a controlled-environment chamber; (ii) provide quantitative data on root-to-shoot ZnO translocation and accumulation in different plant parts; and (iii) contribute to the understanding of the role of soil physical and chemical properties in plant uptake of zinc.

Owing to alkaline nature of soil used in this experiment, even high doses of ZnO were not expected to increase zinc supply in plants. We also assume that barley plants take up zinc mainly from the dissolved phase and that zinc from nano- and microparticles is much less bioavailable.

## 2. Results and Discussion

### 2.1. Characterization of ZnO Nanoparticles and Bulk ZnO

Many of the ZnO properties are related to its crystal structure. ZnO normally forms in the hexagonal (wurtzite) crystal structure with lattice parameters a = 3.25 Å and c = 5.12 Å [11]. However, it has also been found to crystallize in the cubic zinc blende and rock salt structures. The exact shape of crystals of the same mineral can vary, depending on the production method. Therefore, the ZnO powder and the nanoparticle solution were characterized by two different techniques, X-ray powder diffraction and transmission electron microscopy.

**Figure 1:**
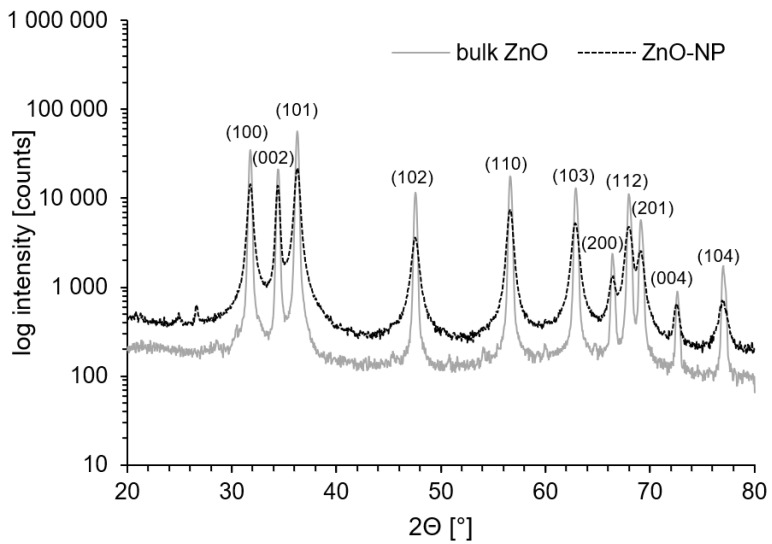
X-ray powder diffraction (XRD) spectrum pattern of nanoparticles (black line) compared to XRD spectrum of bulk ZnO (grey line). The peaks are typical for the mineral zincite with wurtzite structure. A broadening of the diffraction peaks in ZnO-NP diffractogram indicates that investigated material is in nanometer scale.

**Figure 2:**
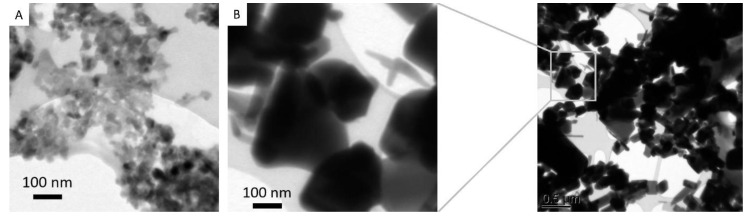
High-resolution transmission electron microscopy images of ZnO (**A**) nanoparticles, (**B**) bulk zincite.

X-ray powder diffraction (XRD) identified the bulk as well as nano-material as zincite (ZnO) with a hexagonal wurtzite-type crystalline structure (Figure 1). Geometrical shapes indicate high crystallinity. Imaging by transmission electron microscope (TEM) was carried out for both ZnO-NP and bulk ZnO to yield information about morphology including particle size, shape and arrangement. Obtained images are presented in Figure 2. The shape of ZnO-NP changes from circular to oval, with some particles possessing edges, like triangles. Nanoparticle size of 10–70 nm was within the size range declared by the manufacturer (below 100 nm). The size and shape of bulk ZnO particles (the conventional ZnO powder) ranged widely from square-and triangular-shaped particles of >500 nm size to smaller spherical particles and elongated, more rod-like particles.

Besides surface chemistry, a nanoparticle size and a physical shape are known to affect the toxicity level. With regard to shape, many studies have found the rod-shaped NPs to elicit a broader range of cytotoxic responses than their equivalent sphere-shaped versions. It is assumed that rods and wires penetrate the cell walls more easily than their spherical counterparts [12]. It is even believed that the genotoxicity of different NPs may primarily be due to particle shape rather than its chemical composition [13,14]. However, the details of how the shape of a nanoparticle influences its toxicity towards cells are still not fully revealed.

### 2.2. Plant Uptake of Zinc as a Reflection of Some Major Soil Properties

The fraction of zinc that is easily exchangeable and potentially bioavailable in soils is very small, being no more than 1%. The most important factors determining zinc availability and toxicity to plants in terrestrial environments are the total zinc content, soil pH, contents of organic carbon, clay, and calcium carbonate, cation exchange capacity, redox conditions, microbial activity in the rhizosphere, soil moisture, concentrations of other trace elements and macronutrients, climate, and the time elapsed from treatment (zinc addition to soil) to toxicity testing. Regarding the latter, it is interesting to note that soils contaminated over a long term generally show reduced toxicity compared to freshly spiked soils [8,15].

Soils and plants are linked by close interactions and any significant change in soil chemical, physical and biological properties is most likely to have an impact on plant growth, development and productivity. Besides crop physiology, morphology and the level of bioavailable fraction of contaminants in the soil, the response of the crop to the application of different amendments also depends on some major soil chemical and physical properties. The textural class of Calcic Chernozem, a soil used in this experiment, was loam with 34% sand, 46% silt and 20% clay. The content of total organic carbon in topsoil was 2.8% and CaCO_3_ content was 3.3%. Soil was classified as moderately alkaline with pH/H_2_O 7.98 and pH/KCl 7.45. Although calcareous soils have been identified as having problems with zinc deficiency, zinc content in this soil was 82.4 mg kg^−1^, which is nearly 30% above the world mean for zinc concentration in soil of 64 mg kg^−1^ as reported by Kabata-Pendias [16]. This was probably due to a low content of CaCO_3_ in surface horizon.

It has already been highlighted in the literature that a high proportion of fine (particularly clay) particles in soil contributes to the retention of zinc in the soil [17,18]. Also, concentrations of zinc in clay and shale parent materials are higher than in other types of rocks. Adsorption mechanisms play an important role in the soil-plant relationships of zinc. These mechanisms control the concentrations of zinc in the soil solution, and hence that which is immediately available to plant roots and also the amounts of zinc in labile forms which can be desorbed and become available to plants. The mechanisms involved in the adsorption of ions on solid surfaces include cation exchange, specific adsorption, binding to organic matter, chemisorption and precipitation [8]. The mechanism that operates in alkaline conditions mainly involves chemisorption and complexation by organic ligands [19]. García-Gómez et al. [20] who investigated the impact of ZnO-NP on various crops cultivated on two soils of different pH and properties believed that the contribution of soil organic matter content to the availability of zinc was small. It appeared to be an influence of pH rather than of organic matter content, because there was variance among the results collected from acidic soil and calcareous soil, the percentage of organic matter was low in both soils and the values were similar (<2%).

It is widely recognized that an important factor influencing the availability of zinc is soil reaction. Generally, zinc is most soluble and hence more available in acid soils. On the other hand*,* less zinc is available in soils with a high pH [21]. The findings of a recently completed experiment carried out by our research team [22] suggest that the attachment of particles and ions to soil along with their size play a crucial role in the distribution and movement of chemical elements in the soil and their availability to soil organisms. By studying the partitioning of zinc in a form of bulk ZnO, ZnO-NP and ZnSO_4_ in suspensions of soils of different types (and thus of different pH) it was shown that ZnSO_4_, representing the ionic form of zinc, adhered better on soil particle surfaces in alkaline soils than in acidic soils; the most alkaline soil tested was Calcic Chernozem and the same soil was used for the purpose of this study. For both ZnO-NP and bulk ZnO the trend was opposite and zinc in these compounds was more prone to be adsorbed onto soil particles when soil pH was acidic. Furthermore, when zinc was applied in the ionic form as ZnSO_4_, it remained dissolved (<1 nm) in all soil solutions across all pH levels tested (4.10–7.98). These findings, along with the results of this study, imply that the impact of particulate ZnO on barley plants was limited due to the fact that a significant portion of zinc that adhered to soil particles and was suspended in soil solution was not completely dissolved and was not necessarily easily available for plant uptake. The results of the study conducted by Šebesta et al. [22] also showed that ZnO-NP were the most mobile of the three tested forms of zinc in alkaline soils.

Generally, zinc content in soils is low compared with that of other essential nutrient elements. Thus, when it comes to zinc, it is more likely that plants are suffering from nutrient deficiency rather than excess. Therefore, most research on zinc in plants has focused on deficiency, which is observed when zinc concentration is less than 10–20 mg kg^−1^, rather than toxicity. Zinc toxicity often occurs in soils affected by mining, smelting and incinerating activities, and in agricultural soils amended with fertilizers, pesticides, manures and sewage sludge; as soil pH falls, zinc solubility and uptake increase and so does a potential for phytotoxicity [23]. While acidic or non-calcareous soils are usually lower in zinc than calcareous ones, the occurrence of deficiency is more common in calcareous than in non-calcareous soils [24]. Calcareous soil is characterized by a high CaCO_3_ content, a high pH value and a low organic matter content, which rank high among factors contributing to zinc deficiency. Although zinc deficiency in crops is widespread and occurs in plants growing in widely varying soil types [24], levels exceeding 300 mg kg^−1^ [25,26] of zinc in dry mass of plant tissue can be harmful to plants. Reaching a level of 500 mg Zn kg^−1^ d. wt. in diagnostic leaves, the affected plants usually exhibit typical phytotoxic symptoms such as reduced dry matter yield [23]. Nonetheless, some more tolerant plant species such as beans and potatoes were reported uninjured by 500 mg Zn kg^−1^ [27], and some, like *Thlaspi caerulescens*, even by doses as high as 40,000 mg Zn kg^−1^ [28].

**Figure 3:**
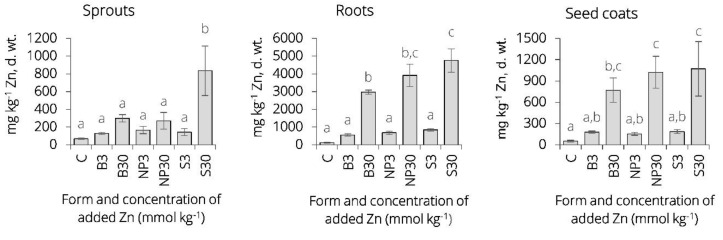
Zinc concentration (mg kg^−1^ d. wt.) in roots, seed coats and sprouts of barley (*Hordeum vulgare* L.) cultivated for seven days in bulk ZnO–, ZnO-NP– and ZnSO_4_–amended soil pots and determined via flame atomic absorption spectrometry (FAAS). Each set of measurements involves sprout/seed coat/root biomass of seven plants ± SD (three replicates). **a**,**b**,**c** letters indicate statistically similar means between the groups as assessed by a one-way ANOVA with Tukey post-hoc test. For characteristics of C, B3, B30, NP3, NP30, S3, S30, see Table 3. Some amount of zinc is passively adsorbed onto the surface of the seeds indicating that only a limited amount of metal is absorbed by the roots and there is a substantial proportion of zinc ions that might be adsorbed onto the root surface. The results also indicate that some differences in Zn concentration in plant tissues, particularly in roots, can be attributed to particle size. During the dissolution of ZnO in the root zone, nanosized particles are absorbed more readily than larger sized particles (bulk ZnO). The plants treated with ZnO-NP show higher concentrations of Zn in roots, although the differences in measured concentrations of Zn in root tissues between the groups treated with ZnO-NP and those treated with bulk ZnO are not significant.

In our experiment, the highest level of zinc content (1200 mg kg^−1^ d. wt.) in leaves was measured in barley grown in pots amended with 30 mmol kg^−1^ of zinc in a form of ZnSO_4_. In general, zinc content in all plant parts was approximately two to twelve times higher in barley grown in soil with 30 mmol Zn kg^−1^ addition than in barley cultivated in soil treated with 3 mmol Zn kg^−1^. As for 300 mg Zn kg^−1^, being the level that has already been associated with toxicity, neither in sprouts nor in seed coats of plants grown in soil treated with 3 mmol Zn kg^−1^ was this value exceeded. However, a physiological response of treated plants was noticeable as described and discussed in more detail in the following section. The results of zinc content analysis were as presented in Figure 3. FAAS analysis confirmed that there was an enhanced zinc translocation into aerial plant parts under zinc treatments at both concentration levels compared to control. A comparison of the plants in the control group with those in the Zn-treated groups with regard to amount of zinc retained in roots and amount of the element that was actually translocated into the above-ground parts suggests that accumulation of zinc in roots may to some extent represent zinc that is adsorbed rather than absorbed.

### 2.3. Impact of Zinc Form and Concentration on Young Barley

Among morphological changes, a size increase or decrease has been the evidence frequently used by researchers for identifying the plant response to the presence of high metal levels; changes in root and shoot length are of those commonly reported. At concentrations of 3 and 30 mmol kg^−1^, ZnO-NP and microparticles of bulk ZnO showed a tendency to negatively influence root growth, which resulted in shortening of the roots (Table 1). However, this negative trend was not statistically significant (α = 0.05). On the other hand, the dissolved form of zinc was found to have a statistically significant negative effect on the root length. In this instance, it would appear that ZnO microparticles (i.e., bulk ZnO) were a zinc form with the least negative impact on young barley plants, followed by ZnO-NP. However, ZnO-NP with a diameter of 10–70 nm did not display statistically significant toxicity either. This trend may be related to increased solubility of ZnO-NP over bulk ZnO not only in acid soils, but also in alkaline ones, and the fact that NPs, given their small size, can penetrate plant cell walls and enter the apoplast. Also, NPs provide larger surface area from which zinc ions can be released simultaneously. Moreover, organic substances, secreted by the plant, dissolve small-size particles more quickly than larger particles. How exactly zinc ions are being released from nanoparticle surface as well as many aspects of Zn (II) movement in plant tissues have, however, not been fully elucidated. In a similar way to our experiment, da Cruz et al. [29] exposed the roots of *Phaseolus vulgaris* L. to ZnO-NP dispersions and ZnSO_4_ to better understand the uptake, biotransformation and physiological response to zinc in bean plants. Near edge X-ray absorption spectroscopy showed that ZnO-NP of 40 nm size were dissolved by roots more easily than their larger-size counterparts.

The toxicity of the dissolved zinc may be mainly related to greater availability of this zinc form for plant roots, where the rate of zinc desorption from soil particles surfaces into soil solution is considerably higher than that of ZnO-NP and bulk ZnO where actual dissolution takes place. Furthermore, zinc in the dissolved form is expected to be distributed somewhat evenly in soil whereas zinc in particulate forms (ZnO-NP and bulk ZnO) may not be able to enter some small pores which are later overgrown by plant roots. Also, ZnO-NP are prone to aggregate due to large surface area and high surface energy [30], thus their impact on the plant is somewhat similar to that of bulk ZnO. Barley sprouts were less affected by three tested zinc forms than the roots. The process of zinc uptake and partitioning in plants is highly controlled, with systems present for sensing and responding to zinc status [31]. Regulatory mechanisms involved in the control of zinc content in plant tissues exist at multiple sites where transport pathways to the aerial parts are present and therefore the aerial part of the plant is usually less affected. Root cells have efficient ion uptake mechanisms. There are also zinc transporters/channels in the Casparian strip, the physical diffusion barrier in root endodermis, which assists in selective uptake of water, nutrients and other ions. Here, the flux of zinc ions entering the xylem is being regulated and so is the amount of zinc transported to the harvestable above-ground parts of the plant. However, in groups treated with higher concentrations of zinc (30 mmol Zn kg^−1^), the length of the sprouts was negatively affected. This negative effect had been predicted and for NP30 and S30 there was a statistically significant change (decrease) in sprout length compared to control, as calculated by Student’s *t*-test (one-tail, α = 0.05). Furthermore, the roots of plants treated with 30 mmol kg^−1^ ZnO-NP were very fragile and more susceptible to breakage. A slightly adverse effect would probably be greater if the experiment lasted longer and plants were grown in zinc-amended soil for more than a week. Possibly, some other processes in plant development may have been affected as well.

The application of zinc in aqueous solution and in particulate form (having particle sizes in the range of <100 nm to >500 nm) at concentration of 3 and 30 mmol Zn kg^−1^ to the soil prior to planting resulted in negatively affected growth parameters (average root length, average shoot length) and average biomass yield; the only exception was the addition of 30 mmol Zn kg^−1^ in the form of bulk ZnO, which had a positive effect on the root growth. A statistically significant reduction in both root length and shoot length was observed only for plants grown in soil spiked with 30 mmol Zn kg^−1^ in the form of ZnO-NP and ionic (dissolved) zinc.

**Figure 4:**
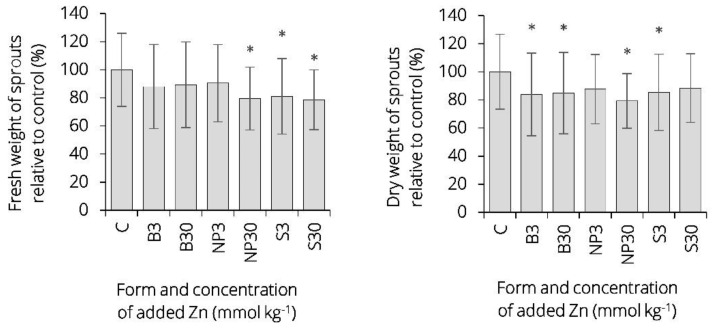
The average relative fresh and dry weight (an average weight of Zn-treated groups compared to the control group) of sprout biomass of barley (*Hordeum vulgare* L.) plants after 10-day cultivation. The data represent mean values ± SD of three independent measurements with seven plants each. Asterisk (*) denotes statistically significant difference between Zn-treated groups and control group on significance level α = 0.05. Zinc was applied in three different forms (bulk, nano-sized, and dissolved) and two concentration levels (3 and 30 mmol Zn kg^−1^). For characteristics of C, B3, B30, NP3, NP30, S3, S30, see Table 3.

A decrease in average sprout fresh weight and total sprout dry weight was observed across all Zn-treated groups compared to control. The weight loss difference between experimental and control group plants amounted to 9–21% for fresh sprout biomass and 11–21% for dry sprout biomass. This weight loss can be attributed to an overall decrease in plant size, particularly root system size. An estimation of below-ground biomass weight was not done due to difficulties encountered during separation of the soil material from the roots. A statistically significant change in sprout fresh weight was observed for NP30, S3 and S30 treatment groups. Regarding relative dry weight of sprouts, a statistically significant change was found for B3, B30, S3 (at the level of 0.05), and NP30 (at the level of 0.01) treatment groups. Relative to the control group, the greatest sprout fresh weight reduction was noticed in S30 and NP30 treatment groups (78.6 and 79.5%, respectively) and the greatest sprout dry weight reduction was recorded for NP30 (79.3%), Figure 4. Relative to the control group, the highest average sprout dry weight was recorded in plants treated with 30 mmol kg^−1^ of zinc in a form of ZnSO_4_ (group S30). In terms of visual toxicity symptoms, barley plants that showed a biomass yield decrease because of excess zinc were stunted but no malformation, discoloration, or necrosis was observed with respect to direct metal toxicity. Recently, barley has been reported amongst the varieties of high-biomass crops that display high metal tolerance [26].

The response of nine crops grown in soils of different pH to ZnO-NP was investigated by García-Gómez et al. [20]. At a concentration range of 225 to 900 mg kg^−1^ ZnO-NP, maize and radish cultivated in calcareous soil responded with an increase in weight relative to control. In contrast, the fresh weight of cucumber and beet grown in the same soil but treated with 450 and 900 mg Zn kg^−1^ and that of wheat treated with 900 mg Zn kg^−1^ decreased. The concentration of available zinc in calcareous soil with pH 8.3 was lower than 1.25 mg kg^−1^ (which made only <0.2% of total Zn) for all experimental groups and this concentration remained rather the same until the harvest; the experiment lasted for 45 days. The authors believe that due to very low level of zinc available, the phytotoxicity was limited to only a slight reduction in the biomass of wheat, cucumber and beet. In acidic soil with pH 5.4, the level of available zinc was high and a significant inhibition in the growth of sprouts was observed for all tested plant species except for pea, over the whole concentration range (20–900 mg Zn kg^−1^). The most dramatic growth suppression was witnessed in radish, tomato, lettuce, cucumber and beet. After 45 days of the experiment, the available fraction of zinc was much higher in acidic soil than in moderately alkaline one because the sorption of zinc on soil components was shown to increase with increasing pH. Nano- and microparticulate materials can interfere with water and nutrient transport in plant [32] and can contribute to enhanced plant water uptake [33]. Therefore, bulk ZnO and ZnO-NP could have had somewhat greater effects on the reduction of dry biomass weight of barley sprouts in spite of higher fresh weight of sprouts in B3, B30 and NP3 treatment groups.

The amounts of chlorophyll a and chlorophyll b in leaves were calculated after measuring the absorbance of the extracts at 625, 647 and 664 nm [34]. In terms of the relative approach, there were no statistically significant changes in the amounts of chlorophyll a and b or in total chlorophyll content across the groups of plants tested (Student’s *t*-test, α = 0.05). The values of chlorophyll b content were less variable than chlorophyll a content values. The concentration of chlorophyll a in leaves was higher than concentration of chlorophyll b. The ratio of chlorophyll a:b, a parameter that indicates the shade tolerance of the species, was relatively constant, varying from 3.43 to 3.80 (Table 2). As for the absolute approach, the ratio of chlorophyll a to b was significantly different only for S30 and B30 group, where the soil was spiked with ZnSO_4_ at 30 mmol Zn kg^−1^. Only the dissolved form of zinc had a little but statistically significant effect on at least one out of four chlorophyll parameters determined. It can therefore be assumed that in alkaline soils zinc in ionic form is somewhat more toxic than its particulate form equivalent.

## 3. Materials and Methods

### 3.1. Site Description, Soil Sampling and Characterization

A loam agricultural soil that was used as a substrate for barley cultivation in the laboratory experiments is typical for the region of Danubian Lowland, which is a part of Little Alföld located in northwest Hungary and extends into southwest Slovakia. This area that spreads between the Danube river, Little Carpathian Mts. and the other parts of Western Carpathians is considered to be the most fertile land in the country. Surface soil (5–15 cm) samples were collected from an arable field in the northwest part of Senec (Slovakia) after removing the upper 5 cm to exclude undecayed and partially decomposed plant remains. The soil was classified according to Morphogenetic Soil Classification System of Slovakia [35] and World Reference Base for Soil Resources [36] as Cernozem kultizemna karbonatova (CMa^c^)/Calcic Chernozem (CH-cc). The soil was then air-dried, ground and sieved (<2 mm) for physical and chemical analyses.

Soil pH was measured potentiometrically using a glass electrode (HI-1230B, Hanna Instruments, Italy) in 1:2.5 soil to solution ratio with deionized water and 1 M KCl solution. CaCO_3_ content in soil was estimated gas-volumetrically using calcimeter apparatus after dissolution of carbonates with 10% HCl. Total organic carbon content was determined following the wet digestion method as described by Walkley and Black [37]. A pipette method was used to determine the soil particle size distribution [38]. The method is based on the difference in sedimentation velocity of particles with different diameters. A known volume of sample was withdrawn by pipette from a given level at predetermined times, the liquid was evaporated off, the solid residue was weighed and the mass percentage of the pipetted fraction was determined. Based on the percentage of sand (2–0.05 mm), silt (0.05–0.002 mm) and clay (˂0.002 mm), soil was classified to textural type by reference to the USDA-FAO soil-texture triangle [39]. Total zinc concentration was determined after decomposition of 1g of soil by acid mixture of HF + HNO_3_ + HclO_4_ + H_2_O_2_ (4:3:4:1, 60 mL) in an open system at 200 °C; sample was then submitted for zinc analysis via FAAS (Perkin-Elmer 1100, Überlingen, Germany).

### 3.2. Preparation of ZnO Nanoparticle Suspensions and ZnSO_4_ Solution

Commercial grade zinc oxide nanoparticles (ZnO-NP) were purchased from Sigma Aldrich, USA (<100 nm particle size (TEM), ≤40 nm avg. part. size (APS), 20 wt. % in H_2_O). Shortly before each experiment, ZnO-NP suspensions with concentration of 9 and 90 mmol Zn L^−1^ were prepared by placing 726 and 7260 µL of the dispersion into 200 mL volumetric flasks that were then adjusted to volume with distilled water. These suspensions were then sonicated for 15 min in an ultrasonic bath.

A 0.1 mmol L^−1^ ZnSO_4_ stock solution was prepared by dissolving 5.751 g of ZnSO_4_·7H_2_O (p.a. quality, CentralChem, Bratislava, Slovakia) in 200 mL of distilled water. Solutions of ZnSO_4_ with concentration of 9 and 90 mmol Zn L^−1^ were prepared by placing 18 and 180 mL of 0.1 mmol L^−1^ ZnSO_4_ stock solution into 200 mL volumetric flasks whose volume was then made up to the mark with distilled water.

### 3.3. Spiking the Soil with Bulk ZnO Powder, ZnO-NP and ZnSO_4_

In the laboratory, seven small plastic containers with a capacity of 0.3 L were filled with 210 g of soil. One pot of soil served as a control (C) with no extra zinc added, the other six were artificially contaminated with Zn (II) in the form of bulk ZnO, ZnO-NP and ZnSO_4_. Specifically, two pots were spiked with bulk ZnO powder (p.a. quality, Chemapol, Prague, Czech Republic); one of them (B3) with 0.0513 g of bulk ZnO while the other (B30) with 0.5130 g of bulk ZnO in order to obtain 3 and 30 mmol Zn kg^−1^ soil, respectively. Approximately 70 mL of distilled water were then added to each pot and the soil was thoroughly mixed. The next two pots were treated with 9 mmol L^−1^ (NP3) and 90 mmol L^−1^ (NP30) dispersions of ZnO-NP, and the last two cups with 70 mL of 9 mmol L^−1^ (S3) and 90 mmol L^−1^ (S30) ZnSO_4_ solutions in order to obtain soil treated with 3 and 30 mmol Zn kg^−1^. All pots were labeled according to the zinc concentration by which they were treated as B3, B30, NP3, NP30, S3, S30 and C (Table 3). Each set of measurements was reproduced three times independently, under the same conditions. The exposure concentrations of 200 and 2000 mg Zn kg^−1^ (i.e., 3 and 30 mmol Zn kg^−1^) were selected based on some reports in the literature [40,41,42]. A dose of 2000 mg kg^−1^ is also commonly reported on the zinc content in swine feed mixture that is a possible route by which zinc is released into the environment or enters the food chain.

### 3.4. Barley Cultivation

Barley seeds were washed in 5 g L^−1^ NaClO and then rinsed two times in distilled water. They were then placed in petri dishes lined with filter paper. During the first three days, the seeds were kept in the dark and watered with 10 mL of distilled water every day. After this period, the newly germinated seedlings were transplanted to polyethylene pots of soil, seven seedlings to each pot, and cultured for another seven days in a controlled environment chamber set at 25 °C, 16 h light/20 °C, 8 h dark cycle. All pots were regularly irrigated with 10 mL distilled water to maintain a proper moisture level. After completion of the cultivation process, plants were carefully removed from soil, washed in distilled water, the lengths of the shoots and the longest root developed were measured and dry weight of plant biomass was recorded after drying the plants at room temperature (25 ± 2 °C) for 7 days. Chlorophyll was extracted from the leaves by *N*,*N*-dimethylformamide (Centralchem, Bratislava, Slovakia). The absorbance was read at 625, 647 and 664 nm with a spectrophotometer (Thermo Scientific Evolution 60S, USA). Calculation of the total content of chlorophyll a and b was done using extinction coefficients and the equations by Moran [34]. Dry shoots, roots and seed coats were separated, weighed and digested for zinc determination via FAAS procedure (Perkin-Elmer 1100, Überlingen, Germany). 0.30 g plant tissue samples were digested in PTFE pressure vessels in the Anton Paar Multiwave 3000 microwave digestion system using 4 mL of concentrated HNO_3_ and 2 mL H_2_O_2_ at 60 bar pressure.

### 3.5. Characterization of ZnO Nanoparticles and Bulk ZnO

To examine the surface morphology and the size of ZnO-NPs and bulk ZnO particles, transmission electron microscope (TEM) images were collected on JEOL-1200 EX instrument (JEOL Ltd., Tokyo, Japan) operating at accelerating voltages of 120 kV. For the analysis, samples were diluted in distilled water and ultrasonicated in order to break up large aggregates. A drop of solution was placed onto a carbon-coated grid and then allowed to dry in air overnight at room temperature. TEM images were acquired at 25–50 kX magnification.

To identify the crystalline phases present in ZnO-NP and bulk ZnO, an X-ray powder diffraction (XRD) analysis has been carried out using Bruker D8 DISCOVER diffractometer (Bruker, Billerica, MA, USA) equipped with X-ray tube with rotating Cu anode operating at 12 kW. All measurements were performed in parallel beam geometry with parabolic Goebel mirror in the primary beam. The X-ray diffraction patterns were recorded in grazing incidence set-up with the angle of incidence α = 2°. The lattice parameter and the crystallite size, in terms of volume weighted column-length, were determined by Pawley method using the software TOPAS 3.0 (Bruker-AXS GmbH, Karlsruhe, Deutschland).

### 3.6. Statistical Analysis

Experimental data were subjected to statistical analysis using Microsoft Excel Data analysis ToolPak (Redmond, WA, USA). An *F*-Test Two-Sample for Variances was run in order to determine whether variances of the control group and variances of the experimental group were statistically equal or not. After performing an *F*-Test, Student‘s *t*-test: Two-Sample Assuming Equal variances (one-tail, α = 0.05) was conducted to examine whether the differences in measured parameters (length or weight reduction) between the control group and Zn-treated groups were statistically significant. For the zinc concentration in sprouts, roots and seed coats, one-way ANOVA with a post-hoc Tukey’s test was run to find if there is a difference in means among the seven treatment groups (C, B3, B30, NP3, NP30, S3, S30).

## 4. Conclusions

Apart from many industrial applications, zinc is used in intensive animal farming and is commonly added to a pig diet at high concentrations although there is potential for negative effects when ZnO products enter the environment via manure. For medicated feeding stuff, literary sources report doses starting at 2000 mg Zn kg^−1^ and so this concentration was selected as our upper-limit concentration value. Since cereals such as barley serve as a staple food for a large part of the world’s population, this cereal grain was tested under varied supply of zinc in soil culture. ZnO nanoparticles and bulk ZnO have been characterized by X-ray powder diffraction and transmission electron microscopy; results confirmed hexagonal wurtzite structure with an average size of 10–70 nm. Physiological response of plants treated with bulk ZnO, nano-sized ZnO and dissolved zinc was evident, the addition of zinc at 3 and 30 mmol kg^−1^ soil eventually resulted in root length shortening. Leaves appeared to be less affected by zinc stress than roots. The relative average weight values of dry sprout biomass that had been exposed to zinc were lower compared to control; the statistically significant adverse effects at the level of 0.05 were observed at concentration of 3 mmol Zn kg^−1^ in bulk ZnO and ZnSO_4_ treated groups, and at 30 mmol Zn kg^−1^ concentration in bulk ZnO (α = 0.05) and nano ZnO (α = 0.01) treated groups. The greatest weight reduction over the control was approximately 21%. FAAS analysis showed a notable difference in zinc concentration in roots and above-ground plant parts. The findings indicate that in order to cope with different environmental conditions, plants have developed a series of mechanisms for efficient uptake of nutrients allowing plants to better adapt to either excess or deficient conditions. The presence of an effective mechanical and/or physiological barrier was also confirmed by the analysis of the chlorophyll content in leaves. Results showed that only at high zinc concentrations, a statistically significant adverse effect of zinc was detected for plants treated with ZnSO_4_, where chlorophyll a/b ratio was significantly lower compared to untreated group. The changes in root and shoot growth and biomass production are also partly a reflection on how soil characteristics affect crop performance under zinc exposure. Although acid pH is often associated with high nanoparticle solubility and an increased ability of nanoparticles to penetrate cells, nano-sized zinc along with two other tested zinc forms were able to enter the roots of barley grown in moderately alkaline soil. It would be desirable to extend this experiment by including more soils of different pH and involving additional soil chemical properties to examine the impact of different soil properties on the availability and toxicity of dissolved and particulate zinc to plants.

## Figures and Tables

**Table 1:** The average length of the longest sprout and root of barley (*Hordeum vulgare* L.) plants, and the average fresh weight and dry weight of sprout biomass after 10-days cultivation. The data represent absolute and relative mean values ± SD of three independent measurements with seven plants each. Zinc was applied in three different forms (bulk, nano-sized, and dissolved) and two concentration levels (3 and 30 mmol Zn kg^−1^). For characteristics of C, B3, B30, NP3, NP30, S3, S30, see Table 3.

Zn Form and Concentration						
C	B3	B30	NP3	NP30	S3	S30
average sprout length (cm)/plant						
17.2 ± 3.3	15.9 ± 3.3	15.6 ± 4.1	16.1 ± 3.5	15.5 ± 2.3 *	16.5 ± 3.3	15.4 ± 3.0 *
average sprout length compared to control (%)						
100.0 ± 16.6	92.7 ±18.0	91.2 ± 23.8	93.7 ± 19.4	91.0 ±15.9	95.9 ±16.3	89.9 ±18.1
average root length (cm)/plant						
9.5 ± 3.1	8.8 ± 3.0	10.2 ± 2.8	8.5 ± 2.8	7.8 ± 2.9 *	8.1 ± 3.1	6.8 ± 2.5 **
average root length compared to control (%)						
100.0 ± 30.2	93.0 ± 29.5	108.2 ± 30.7	89.5 ± 28.6	84.1 ± 32.3	83.2 ± 24.5	71.6 ± 20.8
average sprout fresh weight (mg)/plant						
238.8 ± 65.0	211.1 ± 78.7	212.5 ± 73.1	215.4 ± 67.8	188.5 ± 52.6 **	195.0 ± 70.1 *	186.2 ± 46.3 **
average sprout dry weight d.wt. (mg)/plant						
20.7 ± 5.8	17.4 ± 6.6	17.5 ± 6.0 *	18.1 ± 5.3 *	16.3 ± 4.0 **	17.8 ± 6.1	18.2 ± 4.7

Notes: Standard deviation value was determined from 3 × 7 measurements. * Statistically significant difference between Zn-treated groups and control group on significance level α = 0.05. ** Statistically significant difference between Zn-treated groups and control group on significance level α = 0.01.

**Table 2:** Chlorophyll a and b, total chlorophyll, and chlorophyll a/b ratio. Measurement were performed with 1 inch length specimens cut out of the top of the first and second leaves of seven barley shoots sampled for each treatment group after 10-day cultivation.

	Zn Form and Concentration						
	C	B3	B30	NP3	NP30	S3	S30
	chlorophyll (μg mg^−1^)						
Chl a	1.73 ± 0.26	1.66 ± 0.15	1.52 ± 0.16	1.59 ± 0.14	1.67 ± 0.20	1.64 ± 0.11	1.58 ± 0.22
Chl b	0.45 ± 0.07	0.45 ± 0.04	0.42 ± 0.05	0.42 ± 0.04	0.44 ± 0.06	0.43 ± 0.03	0.46 ± 0.09
Chl total	2.18 ± 0.33	2.11 ± 0.20	1.94 ± 0.21 *	2.01 ± 0.18	2.12 ± 0.26	2.07 ± 0.14	2.04 ± 0.31
a:b	3.80 ± 0.14	3.71 ± 0.13	3.59 ± 0.17 **	3.79 ± 0.09	3.79 ± 0.20	3.80 ± 0.09	3.43 ± 0.22 **
	chlorophyll (%)						
Chl a	100.0 ± 10.0	97.8 ± 15.7	89.4 ± 16.4	92.7 ± 9.4	97.4 ± 9.2	96.2 ± 12.5	92.5 ± 15.6
Chl b	100.0 ± 10.4	99.7 ± 14.0	94.0 ± 13.9	92.9 ± 9.8	97.7 ± 7.6	95.9 ± 11.3	102.5 ± 17.0
Chl total	100.0 ± 10.0	98.2 ± 15.3	90.4 ± 15.8	92.7 ± 9.4	97.5 ± 8.6	96.2 ± 12.2	94.6 ± 15.7
a:b	100.0 ± 3.3	97.7 ± 4.1	94.6 ± 5.1	99.7 ± 2.5	99.7 ± 5.8	100.1 ± 2.6	90.3 ± 6.1

Notes: Standard deviation value was determined from nine measurements. * Statistically significant difference between Zn-treated groups and control group on significance level α = 0.05. ** Statistically significant difference between Zn-treated groups and control group on significance level α = 0.01.

**Table 3:** The concentration of Zn (II) in different forms in replicated pots of soil (21 pots in total).

Pot Label	Soil Treatment	Zn (mg kg^−1^)	Zn (mmol kg^−1^)
C (1–3)	control, no Zn added	0	0
B3 (1–3)	0.0513 mg bulk ZnO, 70 mL of distilled water	200	3
B30 (1–3)	0.5130 mg bulk ZnO, 70 mL of distilled water	2000	30
NP3 (1–3)	ZnO-NP, 70 mL of 9 mmol Zn L^−1^	200	3
NP30 (1–3)	ZnO-NP, 70 mL of 90 mmol Zn L^−1^	2000	30
S3 (1–3)	ZnSO_4_, 70 mL of 9 mmol Zn L^−1^	200	3
S30 (1–3)	ZnSO_4_, 70 mL of 90 mmol Zn L^−1^	2000	30
